# Palm-Based Standard Reference Materials for Iodine Value and Slip Melting Point

**DOI:** 10.4137/aci.s1052

**Published:** 2008-09-22

**Authors:** Azmil Haizam Ahmad Tarmizi, Siew Wai Lin, Ainie Kuntom

**Affiliations:** Analytical and Quality Development Unit, Malaysian Palm Oil Board, 6, Persiaran Institusi, Bandar Baru Bangi, 43000 Kajang, Selangor, Malaysia

**Keywords:** palm-based standard reference materials, iodine value, slip melting point, MPOB Test Methods p3.2: 2004 and p4.2:2004, consensus values, uncertainties

## Abstract

This work described study protocols on the production of Palm-Based Standard Reference Materials for iodine value and slip melting point. Thirty-three laboratories collaborated in the inter-laboratory proficiency tests for characterization of iodine value, while thirty-two laboratories for characterization of slip melting point. The iodine value and slip melting point of palm oil, palm olein and palm stearin were determined in accordance to MPOB Test Methods p3.2:2004 and p4.2:2004, respectively. The consensus values and their uncertainties were based on the acceptability of statistical agreement of results obtained from collaborating laboratories. The consensus values and uncertainties for iodine values were 52.63 ± 0.14 Wijs in palm oil, 56.77 ± 0.12 Wijs in palm olein and 33.76 ± 0.18 Wijs in palm stearin. For the slip melting points, the consensus values and uncertainties were 35.6 ± 0.3 °C in palm oil, 22.7 ± 0.4 °C in palm olein and 53.4 ± 0.2 °C in palm stearin. Repeatability and reproducibility relative standard deviations were found to be good and acceptable, with values much lower than that of 10%. Stability of Palm-Based Standard Reference Materials remained stable at temperatures of −20 °C, 0 °C, 6 °C and 24 °C upon storage for one year.

## Introduction

Palm oil is one of the most important sources of revenue for Malaysia. The total export earnings increased by 41.8% in 2007 compared to that in 2006 (Mohd. Basri, 2008). In fact, palm oil contributes to almost 30% of the vegetable oils production worldwide, in which 60% of the share accounts for the overall global export (Carter et al. 2007). Palm oil has now gained worldwide acceptance due to its unique properties and versatile applications as well as the competitive traded price over other vegetable oils (Choo et al. 2007). As one of the leading countries in the palm oil business, attempts should be taken to ensure that palm oil is of good quality.

Some of the indicators used to characterize palm oil are iodine value (IV) and slip melting point (SMP). The IV measures the degree of unsaturation or double bonds of oils and fats. It also indicates the ease of oxidation of oils and fats (Guided Wave Incorporated, 2008). Meanwhile, the SMP is widely used to characterize the melting and solidification properties of oils and fats. It changes with the chain length of fatty acids, unsaturation ratios, trans fatty acid content and the position of the fatty acids in the glycerol backbone (Karabulut et al. 2004).

Harmonization of the test methods for these characteristics is crucial in producing palm oil within the traded specifications. Thus, a comparison of measurements should be made with the certified values of the standard reference materials to assure the reliability and robustness of the methods used. With the emphasis on the quality aspects, our research group has been engaged in the development of Palm-Based Standard Reference Materials for palm oil analyses due to the unavailability of such standard reference materials in the market. This is to facilitate the use of these standard reference materials for calibration and validation of analytical measurements as well as to assess the capability of analysts in performing the measurements in the palm oil sector.

Currently, Palm-Based Standard Reference Materials for fatty acids composition, solid fat content, IV and SMP have been produced in-house. However, only two characterization works of fatty acids composition and solid fat content have been published. Thus, this paper reports the outcomes of the inter-laboratory proficiency tests of the standard reference materials from palm oil, palm olein and palm stearin for IV and SMP. Stability of the standard reference materials produced upon storage was also evaluated and further discussed in this paper.

## Materials and Methods

### Production of Palm-Based Standard Reference Materials

Refined, bleached and deodorized palm oil, palm olein and palm stearin were obtained from a local supplier. The antioxidant, tert-butylhydroquinone (97% purity) and 5-mL dark amber glass ampoules were purchased from Sigma-Aldrich (Steinheim, Germany) and Scherf Praezision (Meiningen-Dreissigacker, Germany), respectively.

A batch of palm oil, palm olein and palm stearin, respectively were heated up to 70 °C to liquefy the solid fat prior to the addition of 200 mg kg^−1^ of tert-butylhydroquinone. These solutions were then mixed thoroughly to ensure homogeneity. Each batch of oil produced about 3000 ampoules of standards. The usage of 5-mL dark amber glass ampoules helps to avoid color changes and photo-oxidation of oil standards upon storage. Portions of 5 mL of homogenized oils were pipetted into the ampoules prior to flushing with nitrogen and flame sealed. The oil standards were then labelled, packed in fabricated boxes and stored at −20 °C until dispatch.

### Characterization exercises

Thirty-three and thirty-two of local and overseas laboratories collaborated in the inter-laboratory proficiency tests for IV and SMP, respectively, which include Analytical and Quality Development Unit, Malaysian Palm Oil Board, Selangor, Malaysia; Kek Seng Berhad, Johor, Malaysia; Research and Development Laboratory, Golden Jomalina Food Industries Sdn. Bhd., Selangor, Malaysia; Aarhuskarlshamn Sweden AB, Analys-Centrum, Karlshamn, Sweden; PT Multimas Nabati Asahan, Sumatera Utara, Indonesia; Southern Edible Oil Industries Sdn. Bhd., Selangor, Malaysia; Wilmar International Ltd., Singapore; School of Sciences and Food Technology, Universiti Kebangsaan Malaysia, Selangor, Malaysia; Quality Assurance Laboratory, Golden Jomalina Food Industries Sdn. Bhd., Selangor, Malaysia; PT Asianagro Agungjaya, West Java, Indonesia; Kempas Edible Oils Sdn. Bhd., Johor, Malaysia; PGEO Edible Oils Sdn. Bhd., Johor, Malaysia; IOI Edible Oils Sdn. Bhd., Sabah, Malaysia; Oleochemical Products Services Unit, Malaysian Palm Oil Board, Selangor, Malaysia; Pan-Century Edible Oils Sdn. Bhd., Johor, Malaysia; Edtech Associates Sdn. Bhd., Penang, Malaysia; Chemara Laboratory Sdn. Bhd., Negeri Sembilan, Malaysia; ITS Testing Services Sdn. Bhd., Selangor, Malaysia; Kuala Lumpur-Kepong Berhad, Selangor, Malaysia; Biochem Laboratories Sdn. Bhd., Penang, Malaysia; Allied Chemists Sdn. Bhd., Johor, Malaysia; Felda-Johore Bulkers Sdn. Bhd., Johor, Malaysia; Chemsain Konsultant Sdn. Bhd., Sarawak, Malaysia; Lotus Laboratory Services Sdn. Bhd., Johor, Malaysia; SGS Laboratory Services Sdn. Bhd., Johor, Malaysia; Alami Technological Services Sdn. Bhd., Selangor, Malaysia; Chemical Laboratory Sdn. Bhd., Johor, Malaysia; Kuala Lumpur-Kepong Edible Oils Sdn. Bhd. (East Malaysia), Sabah, Malaysia; Testing Services (Sabah) Sdn. Bhd., Sabah, Malaysia; MM Vitaoils Sdn. Bhd., Selangor, Malaysia; PGEO Edible Oils Sdn. Bhd. (Prai Division), Penang, Malaysia; KL-Kepong Edible Oils Sdn. Bhd., Johor, Malaysia; Lam Soon Edible Oils Sdn. Bhd., Johor, Malaysia.

Four ampoules of each oil standards (palm oil, palm olein and palm stearin), which correspond to four replications, were sent to the collaborating laboratories. The IV and SMP measurements should be conducted using MPOB Test Methods p3.2:2004 and p4.2:2004 (Ainie et al. 2004), respectively. The MPOB Test Methods p3.2:2004 was technically equivalent to ISO 3961:1996. The MPOB Test Methods p4.2:2004 was originated from AOCS Official Method Cc 3–25 (Firestone, 1998).

In the determination of IV, about 0.2 g of oil sample, in a 20 mL mixture of cyclohexane (Merck, Darmstadt, Germany) and glacial acetic acid (Systerm, Shah Alam, Malaysia) was reacted with 25 mL of Wijs reagent (Merck, Darmstadt, Germany) followed by addition of 20 mL of 100 g L^−1^ potassium iodide (Systerm, Shah Alam, Malaysia) and distilled water after storage in the dark for 1 h. The liberated iodine was titrated with 0.1 M sodium thiosulfate (Univar, Seven Hills, Australia) until the yellowish iodine color disappeared. A small amount of starch solution (Merck, Darmstadt, Germany) was then added to the solution as an indicator and the titration continued until the blue color has also disappeared. The IV is calculated by the following equation :

$$IV(g/100g)=\frac{12.69C(V_{1}-V_{2})}{m}$$

where,

*C*: is the concentration of sodium thiosulfate solution (mole L^−1^);
*V*_1_: is the volume (mL) of sodium thiosulfate solution used for the blank test;
*V*_2_: is the volume (mL) of sodium thiosulfate solution used for the sample; and
*m*: is the mass (g) of the sample

Wijs reagent, which contains iodine monochloride in acetic acid, can be also prepared manually in the laboratory. The ratio between iodine and chlorine of the Wijs reagent shall be within the limits of 1.10 ± 0.1. However, the preparation of the reagent is time consuming and hence is highly recommended to use the commercially available Wijs reagent.

SMP was measured using the following procedure. At least three clean capillary tubes were initially dipped into a completely melted oil sample to a depth of 10 mm. The tubes were then chilled until the oil sample was solidified prior to placing them in a test tube and held in a beaker of water equilibrated at 10 °C for 16 h in a thermostat water bath (Huber, Offenburg, Germany). The capillary tubes were subsequently removed from the test tube and attached to a thermometer with a rubber band such that the lower ends of the tubes were at the same level as the bottom of the mercury bulb of the thermometer. The thermometer was suspended in a beaker containing 400 mL of boiled distilled water. The thermometer should be immersed in the water to a depth of 30-mm. The initial temperature of the thermostat water bath was adjusted between 8 to 10 °C below the expected SMP of the oil sample. The water bath was agitated using a magnetic stirrer and heat was supplied at the rate of 1 °C min^−1^ and reduced to 0.5 °C min^−1^. The temperature at which the sample in the tubes started to melt and become clear is defined as the SMP. The difference between values of the measurement carried out by the same analyst on the same test sample shall not exceed 0.8 °C for palm oil and 0.5 °C for palm olein and palm stearin.

Apart from the oil standards, each collaborator was also supplied with detailed instructions of the study protocol and reporting cards to compute their analysis results. The IV and SMP analyses should be carried out within two month’s time before sending out the results to the Malaysian Palm Oil Board.

### Statistical evaluation

Inter-laboratory proficiency tests results were assessed using the SoftCRM Version 1.2.0 software (Bonas, 1997). The software is particularly applied to evaluate the standard reference materials data as well as to document the quality of the standard reference materials produced. Consensus values for IV and SMP of each oil standard were generated at 95% confidence interval (CI). Outlying data or extreme values were discarded based on Grubb and Cochran tests. Grubb test determines outlying mean values (variability) between laboratories, whereas Cochran test identifies data variability within laboratories (Pocklington and Wagstaffe, 1987; ISO 5725–2 1994; Azmil Haizam et al. 2008).

Repeatability relative standard deviation, RSD_r_ (relative standard deviation within laboratory) and reproducibility relative standard deviation, RSD_R_ (relative standard deviation between laboratories) were also calculated for IV and SMP (ISO 5725–2 1994). The RSD_r_ is determined from the test results generated under repeatability conditions of the same method on identical test items in the same laboratory by the same analyst using the same instrument within short intervals of time. Meanwhile RSD_R_ is identified from the test results generated under reproducibility conditions in which the results are obtained using the same method on identical test items in different laboratories and analysts using different instruments (ISO 3541–1 1993).

### Stability test

Stability of the Palm-Based Standard Reference Materials was monitored for 12 months at four storage conditions of −20 °C, 0 °C, 4 °C and 24 °C. The oil standards were randomly analyzed for their IV and SMP at predetermined storage intervals.

## Results and Discussion

### Characterization of lodine value

**Figure 1.**
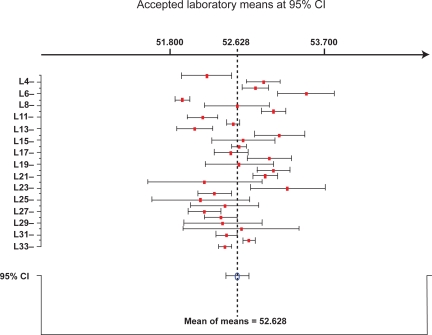
Example of bar graph for characterising iodine value in palm oil. The results plotted correspond to four replications. Mean of means designates the average result of total individual laboratory means at 95% confidence interval (CI).

Statistical evaluations for IV in palm oil, palm olein and palm stearin are tabulated in Table 1. The consensus values (IV) and their uncertainties were calculated at 95% CI using the SoftCRM 1.2.0 software. Data of thirty laboratories were retained for IV characterization in all palm products, while only three laboratories were discarded. The RSD_r_ of the characterized IV in the three oils ranged from 0.40 to 0.64%, while RSD_R_ of 0.79%, 0.64% and 1.50% was achieved in palm oil, palm olein and palm stearin, respectively. Both RSD_r_ and RSD_R_ were observed to be more than 5-fold lower than that of 10%, which signified that the IV analysis performed was within the acceptable variability. Example of intra- and inter- laboratory variability of IV in palm oil is illustrated in Figure 1. The bar graph consists of laboratory codes with their individual means and standard deviations after elimination of outliers through Cochran and Grubb tests at 95% CI. From that, the overall mean of means and its standard deviation was then determined from the accepted values. All the values were obtained according to ISO Guide 35 (1989).

### Characterization of slip melting point

**Figure 2.**
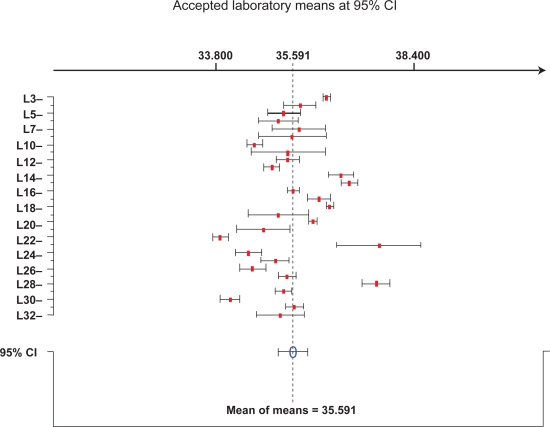
Example of bar graph for characterising slip melting point in palm oil. The results plotted correspond to four replications. Mean of means designates the average result of total individual laboratory means at 95% confidence interval CI.

Table 2 summarizes the statistical evaluations of SMP in palm oil, palm olein and palm stearin. The RSD_r_ and RSD_R_ were also identified for the characterized SMP. Three out of 32 laboratories were eliminated for characterization of SMP in palm oil while 25 and 26 laboratories were retained in palm olein and palm stearin, respectively. In general, both RSD_r_ and RSD_R_ were found to be less than 10%. The RSD_r_ of palm oil, palm olein and palm stearin was 0.76%, 0.59% and 0.27%, respectively. These values were almost comparable with that of RSD_r_ for IV characterization. The RSD_R_, however, exhibited slightly higher values for palm oil (2.57%) and palm olein (4.08%) in comparison to that of IV characterization. The higher RSD_R_ is generally due to run-to-run variations, which may be caused by the variation in the instrument sensitivity, change of environmental conditions or even uncontrolled change of instrument parameters (Azmil Haizam et al. 2007; Azmil Haizam et al. 2008). Such effects are important, which could influence the RSD_R_ when the samples were analysed by different laboratories. Only palm stearin produced a lower RSD_R_ of 0.87%. Furthermore, both RSD_r_ and RSD_R_ were still well below 10% and hence the SMP results were acceptable. Similar to IV, the SMP assessment can also be expressed in the form of bar graphs. Figure 2 shows an example of within- and between- laboratory variability of SMP in palm oil.

### Stability of Palm-Based Standard Reference Materials

Results of storage stability tests for IV and SMP are showed in Table 3 and Table 4, respectively. The test results of the four storage conditions (−20 °C, 0 °C, 4 °C and 24 °C) were compared with oil standards that were initially prepared and stored at −20 °C (t = 0). No detectable changes of IV and SMP were perceived even at 24 °C. As the Palm-Based Standard Reference Materials of these characteristics were observed to have good stability, their shelf-life could be extended for more than one year.

## Conclusion

From the characterization exercises, it can be concluded that the production of Palm-Based Standard Reference Materials (palm oil, palm olein and palm stearin) for iodine value and slip melting point is achievable through inter-laboratory proficiency tests. The establishment of the consensus values (certified values and their uncertainties) at an acceptable level of 95% confidence interval has been attained using the SoftCRM 1.2.0 software. Consensus values of these Standard Reference Materials remained stable after one year of storage and hence their shelf-life could be prolonged for another one year.

## Figures and Tables

**Table 1.** Statistical evaluation of iodine value in Palm-Based Standard Reference Materials.

**Standards**	**P^a^**	**N^b^**	**Consensus value (Uncertainty)^c^**	**s_r_^d^**	**RSD_r_^e^**	**R^f^**	**S_R_^g^**	**RSD_R_^h^**	**R^i^**
Palm Oil	30	120	52.63 (0.14)	0.21	0.40	0.58	0.42	0.79	1.16
Palm Olein	30	120	56.77 (0.12)	0.17	0.31	0.49	0.37	0.64	1.02
Palm Stearin	30	120	33.76 (0.18)	0.22	0.64	0.61	0.51	1.50	1.41

^a^Number of laboratories retained after eliminating outliers.

^b^Number of accepted test results (replicates).

^c^Consensus value and uncertainty (Wijs) generated as 95% confidence interval.

^d^Repeatability standard deviation.

^e^Repeatability relative standard deviation.

^f^Repeatability limit.

^g^Reproducibility standard deviation.

^h^Reproducibility relative standard deviation.

^i^Reproducibility limit.

**Table 2.** Statistical evaluation of slip melting point in Palm-Based Standard Reference Materials.

**Standards**	**P^a^**	**N^b^**	**Consensus value (Uncertainty)^c^**	**s_r_^d^**	**RSD_r_^e^**	**R^f^**	**S_R_^g^**	**RSD_R_^h^**	**R^i^**
Palm Oil	29	116	35.6 (0.3)	0.27	0.76	0.76	0.91	2.57	2.56
Palm Olein	25	100	22.7 (0.4)	0.13	0.59	0.38	0.93	4.08	2.60
Palm Stearin	26	104	53.4 (0.2)	0.14	0.27	0.40	0.46	0.87	1.30

^a^Number of laboratories retained after eliminating outliers.

^b^Number of accepted test results (replicates).

^c^Consensus value and uncertainty (°C) generated as 95% confidence interval.

^d^Repeatability standard deviation.

^e^Repeatability relative standard deviation.

^f^Repeatability limit.

^g^Reproducibility standard deviation.

^h^Reproducibility relative standard deviation.

^i^Reproducibility limit.

**Table 3.** Changes of iodine value in Palm-Based Standard Reference Materials.

**Standards**	**T^a^(°C)**	**Storage period (month)^b^**								
		**0**	**2**	**4**	**6**	**8**	**10**	**12**	**Mean**	**SD^c^**
Palm Oil	−20	52.25 ± 0.19	52.65	52.55	52.15	52.55	52.40	52.50	52.47	0.18
	0		52.55	52.60	51.90	52.20	52.40	52.70	52.39	0.30
	6		52.60	52.60	51.70	52.45	52.50	52.55	52.40	0.35
	24		52.70	52.60	52.05	52.45	53.30	52.60	52.62	0.41
Palm Olein	−20	57.20 ± 0.08	56.80	56.65	56.15	56.20	56.25	55.40	56.24	0.49
	0		57.00	56.70	56.25	56.55	56.00	56.00	56.42	0.40
	6		56.80	56.80	56.15	56.60	56.20	56.15	56.45	0.32
	24		56.05	56.45	56.20	56.65	56.10	55.90	56.23	0.28
Palm Stearin	−20	34.18 ± 0.32	33.90	34.70	33.70	34.10	34.20	34.20	34.13	0.34
	0		33.95	34.70	33.40	34.20	34.30	34.20	34.13	0.43
	6		34.10	34.70	33.65	34.20	34.15	34.25	34.18	0.34
	24		33.90	34.70	33.90	34.20	34.30	34.30	34.22	0.30

^a^Storage temperature.

^b^Mean of duplicate (Wijs).

^c^Standard deviation.

**Table 4.** Changes of slip melting point in Palm-Based Standard Reference Materials.

**Standards**	**T^a^ (°C)**	**Storage period (month)^b^**									
		**0**	**2**	**4**	**6**	**8**	**10**	**12**	**Mean**	**SD^c^**
Palm Oil	−20	36.4 ± 0.1	36.4	36.3	35.7	36.0	35.8	36.1	36.05	0.27
	0		36.1	35.8	36.1	35.8	36.0	35.9	35.95	0.14
	6		36.0	36.3	35.9	36.0	35.9	36.3	36.07	0.19
	24		36.2	35.8	36.0	35.7	36.2	36.0	35.98	0.20
Palm Olein	−20	22.4 ± 0.2	22.4	22.4	22.9	22.6	23.1	22.3	22.62	0.32
	0		22.7	22.5	22.4	23.0	22.6	22.8	22.67	0.22
	6		22.5	22.9	23.0	23.2	22.5	22.6	22.78	0.29
	24		22.4	22.2	22.8	23.0	22.8	23.1	22.72	0.35
Palm Stearin	−20	53.2 ± 0.1	53.4	53.1	53.0	53.4	53.0	53.2	53.18	0.18
	0		53.0	52.9	53.4	53.5	53.3	54.0	53.35	0.39
	6		53.2	53.4	53.7	53.1	53.0	52.9	53.22	0.29
	24		53.4	53.3	53.2	53.1	53.5	53.0	53.25	0.19

^a^Storage temperature.

^b^Mean of duplicate (°C).

^c^Standard deviation.
